# Hair-cortisol and hair-BDNF as biomarkers of tinnitus loudness and distress in chronic tinnitus

**DOI:** 10.1038/s41598-022-04811-0

**Published:** 2022-02-04

**Authors:** Laura Basso, Benjamin Boecking, Patrick Neff, Petra Brueggemann, Eva M. J. Peters, Birgit Mazurek

**Affiliations:** 1 Tinnitus Center, Charité – Universitätsmedizin Berlin, Berlin, Germany; 2 Department of Psychiatry and Psychotherapy, University of Regensburg, Regensburg, Germany; 3 University Research Priority Program ‘Dynamics of Healthy Aging’, University of Zurich, Zurich, Switzerland; 4 Department of Psychology, Centre for Cognitive Neuroscience, University of Salzburg, Salzburg, Austria; 5 Psychoneuroimmunology Laboratory, Department of Psychosomatic Medicine and Psychotherapy, Justus-Liebig University Giessen, Giessen, Germany; 6 Psychosomatics and Psychotherapy, Charité Center 12 Internal Medicine and Dermatology, Charité – Universitätsmedizin Berlin, Berlin, Germany

**Keywords:** Psychology, Biomarkers, Diseases, Endocrinology, Medical research

## Abstract

The role of stress and its neuroendocrine mediators in tinnitus is unclear. In this study, we measure cortisol as an indicator of hypothalamus–pituitary–adrenal (HPA) axis alterations and brain-derived neurotrophic factor (BDNF) as a marker of adaptive neuroplasticity in hair of chronic tinnitus patients to investigate relationships with tinnitus-related and psychological factors. Cross-sectional data from chronic tinnitus inpatients were analyzed. Data collection included hair sampling, pure tone audiometry, tinnitus pitch and loudness matching, and psychometric questionnaires. Elastic net regressions with n-fold cross-validation were performed for cortisol (N = 91) and BDNF (N = 87). For hair-cortisol (R^2^ = 0.10), the strongest effects were sampling in autumn and body-mass index (BMI) (positive), followed by tinnitus loudness (positive) and smoking (negative). For hair-BDNF (R^2^ = 0.28), the strongest effects were hearing aid use, shift work (positive), and tinnitus loudness (negative), followed by smoking, tinnitus-related distress (Tinnitus Questionnaire), number of experienced traumatic events (negative), and physical health-related quality of life (Short Form-12 Health Survey) (positive). These findings suggest that in chronic tinnitus patients, higher perceived tinnitus loudness is associated with higher hair-cortisol and lower hair-BDNF, and higher tinnitus-related distress with lower hair-BDNF. Regarding hair-BDNF, traumatic experiences appear to have additional stress-related effects, whereas hearing aid use and high physical health-related quality of life appear beneficial. Implications include the potential use of hair-cortisol and hair-BDNF as biomarkers of tinnitus loudness or distress and the need for intensive future research into chronic stress-related HPA axis and neuroplasticity alterations in chronic tinnitus.

## Introduction

The pathogenic mechanisms linking tinnitus and stress are still not fully understood^1–3^. Stress can be related to the onset of tinnitus, and higher stress levels seem associated with higher perceived tinnitus severity^1,2^. Recently, the need to distinguish between tinnitus perception (symptom) and tinnitus associated with suffering (tinnitus disorder) has been highlighted^4^. Moreover, perceived tinnitus loudness and tinnitus-related distress are two distinct phenomena^5^ that appear linked by psychological factors like tinnitus acceptance^6^ and subjective stress level^7^.

The stress response refers to an organism's reactive or anticipatory response to acute challenges^8^ and, in the short term, is an adaptive process to maintain homeostasis^9^. Chronic stress, however, can lead to maladaptation with long-term pathophysiological effects, often described by the concept of allostatic load/overload^9^. Emotional exhaustion, resulting from chronic stress, was found to mediate the relationship between hearing loss and tinnitus severity^10,11^. Moreover, common psychological conditions in tinnitus patients, such as anxiety^12^ and depression^13^, are known to be chronic stress-related^11,14^. Overall, chronic stress is an important factor in tinnitus patients seeking clinical help.

The hypothalamus–pituitary–adrenal (HPA) axis is a primary neuroendocrine stress response system. Chronic stress can lead to a dysregulation of the HPA axis, which can manifest in altered stress response profiles to acute challenges^8^. Previous studies on HPA axis function measuring salivary cortisol in tinnitus patients reported lower overall cortisol levels^15^, a blunted cortisol response to an acute experimental psychosocial stressor^16^, increased sensitivity of the HPA axis negative feedback response found with the dexamethasone suppression test^17^, and a flattened cortisol awakening response in tinnitus patients with high distress^18^. While these studies on salivary cortisol indicate reduced responsiveness of the HPA axis in tinnitus, findings on blood-cortisol levels are conflicting, reporting no association^19^, negative associations with tinnitus frequency and loudness^20^, or treatment-related decreases^21^. Overall, HPA axis alterations in tinnitus and their relationship with tinnitus-related distress remain unclear.

Saliva and blood sampling are methods for the measurement of short-term cortisol release boosts, which can be influenced by situational factors^22,23^. By contrast, the measurement of cortisol in hair provides a reliable long-term measure of cumulative cortisol secretion, reflecting integrated HPA axis activity^24–26^. Given the average growth rate of hair by 1 cm per month^27^, analysis of the 1 cm hair segment most proximal to the scalp allows a retrospective estimate of cumulative cortisol production over the past month. A meta-analysis aggregating data from 66 studies found that different groups exposed to chronic stress (e.g., caregiving stress, unemployment, natural disasters) showed overall elevated hair-cortisol levels by 22% compared to controls^22^.

Brain-derived neurotrophic factor (BDNF) is another important stress-related biomarker and a crucial factor for neuroprotection and synaptic plasticity^28^. Several neurodegenerative and psychiatric disorders are associated with reduced BDNF levels in both the blood and the brain^29,30^. It is thought that acute stress increases BDNF levels, whereas chronic stress leads to a downregulation of BDNF^31,32^. The mixed results of previous studies on BDNF levels in tinnitus patients^33–36^ might be related to situational influences on BDNF measurement in blood. BDNF concentrations can also be measured in hair, which was shown in a pilot study^37^. Measurement of hair-BDNF may offer a new approach to clarify the long-term neuroendocrine changes in chronic tinnitus and associations of BDNF with tinnitus-related distress.

In addition to the sampling material, another important issue is the handling of confounders, as both stress-related biomarkers and tinnitus characteristics can be influenced by a multiplicity of factors. The presence of many and correlated variables is associated with variable selection problems for multivariable regression modeling, which can cause selection bias, overfitting, and replication issues^38^. Elastic net regression is a modern penalized regression procedure that addresses these issues. It counteracts collinearity and overfitting by introducing a penalty term and selection bias by performing automatic variable selection^38,39^.

To date, hair-cortisol and hair-BDNF have not been studied in tinnitus. This study investigates hair-cortisol and hair-BDNF in chronic tinnitus patients and their associations with tinnitus-related and psychological factors while adequately controlling for confounding influences by elastic net regression. The main aim of the present study is to identify relationships of these biomarkers with tinnitus-related distress, as this might provide instructive new insights into their potential use as therapeutic efficacy measures. Biomarker measurement in hair and the use of this state-of-the-art methodological approach represent the strengths that set our study apart from previous research. Based on assumed long-term stress-related effects in chronic tinnitus patients, our hypotheses are that increased hair-cortisol levels and decreased hair-BDNF levels are associated with higher tinnitus-related distress.

## Methods

In total, 94 chronic tinnitus patients volunteered to participate in this study (approx. 16% of treated inpatients) between December 2018 and March 2020 (data collection was stopped due to the COVID-19 pandemic). Inclusion/exclusion criteria are shown in Table 1. Of the recruited patients, one was excluded due to missing data on all questionnaires (hair sample not analyzed), two patients were excluded due to the hair-related criteria, and four patients were excluded due to missing BDNF values. Thus, the final sample consisted of N = 91 for cortisol and N = 87 for BDNF analyses. All participants were European, around two-thirds of the sample were female (65.9%) and participants’ age ranged from 19 to 80 years (M = 51.5, SD = 12). All participants provided written informed consent. The study was approved by the local ethic commission of the Charité – Universitätsmedizin Berlin (No. EA1/035/16) and was carried out in accordance with the Declaration of Helsinki.

**Table 1:** Inclusion and exclusion criteria.

Inclusion criteria	Exclusion criteria
Diagnosis of chronic subjective tinnitus	Inability to consent due to serious mental or physical impairments
Age ≥ 18 years	Simultaneous participation in other research studies
Written informed consent	Hair length < 3 cm
	Any chemical hair treatment within 1 month prior to sampling (dying, bleaching, perming, or else)
	Hair washing or the use of hair products (hair mousse, hair gel, hair wax, hair spray) within 3 days prior to sampling
	Hair combing on the day of sampling

Cross-sectional measurements included the collection of hair samples and the completion of psychometric questionnaires on the same day and were performed at the Tinnitus Center. In addition, audiometric data were obtained from outpatient audiometric records. On average, audiometric testing was performed 70.14 days (SD = 57.62) prior to the other measurements.

### Hair sampling

#### Sample collection

The median sampling time was 09:55 a.m. Samples were cut from the region of the posterior vertex as close to the scalp as possible. Scissors and other materials were cleaned with 70% isopropyl alcohol between samples. Samples were wrapped in aluminum foil and stored at room temperature in a dark container. All samples were analyzed in summer/autumn 2020 (around 1.75 to 0.5 years after collection).

#### Cortisol and BDNF extraction and detection

Hair sample analyses for the detection of cortisol and BDNF in the 1-cm hair segment closest to the scalp followed the previously published laboratory protocol detailed in^37^. Briefly summarized, it included the following steps: (1) segmentation and weighting (5–20 mg), (2) pulverization using a ball mill, (3) extraction procedures for cortisol (1 ml methanol per 10 mg pulverized hair, incubation, centrifugation, drying) and BDNF (220 μl citric acid per 10 mg pulverized hair, centrifugation, lyophilization), (4) quantification using ELISA following manufacturer instructions. According to the manufacturer, the intra- and inter-assay coefficients of variation are + 3.7% and + 8.5% for BDNF ELISA; and + 4.3% and + 13.2% for cortisol ELISA, respectively. In our study, the intra- and inter-assay coefficients of variation were + 2.73% and 5.31 ± 3.35 for BDNF; and + 1.91% and 7.49 ± 2.81 for cortisol. All but four BDNF values were inside the detection range.

### Psychometric questionnaires

German versions of the following psychometric questionnaires were used.

#### Tinnitus questionnaire (TQ)

The Tinnitus Questionnaire^40^ is an instrument assessing tinnitus-related distress. It consists of 52 questions rated on a three-point Likert scale. Only the total score (sum over 40 items, with two items added twice) was used; for sample description, it was categorized based on clinical cut-off scores^40^. Cronbach’s alpha = 0.94.

#### Perceived stress questionnaire (PSQ-20; 20 item version)

The Perceived Stress Questionnaire^41,42^ measures subjectively experienced stress and consists of 20 items rated on a four-point Likert scale. In the present study, the period surveyed has been changed from last month to last week. Only the total score (linear transformed mean over all items) was used; for sample description, it was categorized based on clinical norms from healthy adults^42^. Cronbach’s alpha = 0.93.

#### Hospital anxiety and depression scale (HADS)

The Hospital Anxiety and Depression Scale^43,44^ measures levels of anxiety and depression in the past week and consists of 14 items rated on a four-point Likert scale. The sum scores for anxiety and depression were used (comprising 7 items each); for sample description, they were categorized based on clinical cut-off scores^43^. Cronbach’s alpha: anxiety = 0.79; depression = 0.81.

#### Screening of somatoform disorders (SOMS; 7 days version)

The Screening of Somatoform Disorders (“Screening für somatoforme Störungen”)^45^ is an instrument for recording somatoform symptoms, i.e., medically unexplained physical symptoms affecting the subject's well-being. The questionnaire consists of a list of symptoms (52 symptoms for women; 48 for men) and respondents are asked to indicate whether they have suffered from these symptoms in the last 7 days and rate the degree of associated impairment. The number of reported symptoms was used.

#### State-trait anxiety inventory (STAI): state anxiety

The scale of the State-Trait Anxiety Inventory (STAI)^46^ which measures the current state anxiety (form X1) was used. The scale consists of 20 items on a four-point Likert scale, which were summed to form the total score. Cronbach’s alpha = 0.93.

#### Posttraumatic diagnostic scale (PDS): event list

The traumatic event list of the Posttraumatic Diagnostic Scale (PSD)^47^ was used to assess whether respondents had experienced relevant traumatic events in their past. It consists of 12 items reflecting highly stressful or traumatic experiences. For each event, respondents indicate whether they have experienced it (personally or as a witness). The number of experienced traumatic events was used.

#### Short form-12 health survey (SF-12)

The Short Form-12 Health Survey (“Fragebogen zum Gesundheitszustand”)^48,49^, version 2, was used for the assessment of health-related quality of life. It consists of 12 items on a three- or five-point Likert scale. T-standardized scale values were calculated for the physical component summary and mental component summary using normative data for scoring^49^; for sample description, they were dichotomously categorized (using 1 SD below average as cut-off value). Cronbach’s alpha: physical component summary = 0.89; mental component summary = 0.87.

### Tinnitus and hearing

#### Pure tone audiogram

Pure tone audiometry data were collected from outpatient audiometric records. Hearing thresholds had been measured for the frequencies from 0.25 to 8 kHz and were collected in 5-decibel (dB) intervals for each ear. The average hearing threshold (dB) at all frequencies (0.25, 0.5, 1, 2, 3, 4, 6, and 8 kHz) was calculated for each ear, and further averaged across both sides (if possible). For sample description (Table 2), the mean hearing threshold was categorized by severity^50^.

**Table 2:** Sample characteristics (N = 91).

Variable	Mean (SD)/percentage (N)
**Biomarkers**	
Hair-cortisol (µg/dl)	0.054 (0.047)
Hair-BDNF (ng/ml); N = 87	77.81 (27.56)
**Sociodemographic information**	
Sex	Female: 65.9% (60)
Age	51.5 (12.0)
Marital status	Single: 28.6% (26) Cohabiting or married: 53.8% (49) Separated or divorced or widowed: 17.6% (16)
Education level^a^	Low: 16.5% (15) Medium: 35.2% (32) High: 48.4% (44)
Employment	Yes: 74.7% (68)
**Psychometric questionnaires**	
TQ total score: tinnitus-related distress	36 (16)
	Mild (0–30): 39.6% (36) Moderate (31–46): 37.4% (34) Severe (47–59): 12.1% (11) Very severe (60–84): 11.0% (10)
PSQ-20 total score: perceived stress	51.2 (18.8)
	Normal (≤ 50): 49.5% (45) Mild (51–66): 27.5% (25) Moderate (67–83): 22.0% (20) Severe (≥ 84): 1.1% (1)
HADS: anxiety	8 (4.1)
	Normal (0–7): 47.3% (43) Mild (8–10): 20.9% (19) Moderate (11–14): 28.6% (26) Severe (15–21): 3.3% (3)
HADS: depression	6.1 (3.8)
	Normal (0–7): 60.4% (55) Mild (8–10): 27.5% (25) Moderate (11–14): 12.1% (11) Severe (15–21): 0% (0)
SOMS: somatization	9.6 (7.1)
STAI total score: state anxiety	45.2 (11.3)
PDS: number of traumatic experiences	1.7 (1.4)
SF-12: physical component summary	41.9 (10.1)
	Normal/average (≥ 40): 60.4% (55) Impairments (< 40): 37.4% (34) Missing: 2.2% (2)
SF-12: mental component summary	37.5 (10.1)
	Normal/average (≥ 40): 41.8% (38) Impairments (< 40): 56.0% (51) Missing: 2.2% (2)
**Tinnitus and hearing**	
Matched tinnitus frequency (Hz)	5386.4 (2424.3) Missing: 27.5% (25)
Matched tinnitus loudness (dB)	39.1 (19.8) Missing: 27.5% (25)
Tinnitus: course?	Intermittent: 58.2% (53) Constant: 41.8% (38)
Tinnitus: onset associated with stress?	Yes: 49.5% (45)
Tinnitus: influenced by stress?	Yes: 79.1% (72)
Hyperacusis (self-report)	Yes: 80.2% (73)
Mean hearing threshold (dB)^b^	22.7 (13.0)
	No impairment (≤ 25 dB): 62.6% (57) Mild/slight impairment (26–40 dB): 30.8% (28) Moderate impairment (41–60 dB): 4.4% (4) Severe impairment (61–80 dB): 2.2% (2) Profound impairment (≥ 81 dB): 0% (0)
Use of hearing aids	Yes: 17.6% (16)
**Covariates**	
Season of sample collection	Winter: 48.4% (44) Spring: 16.5% (15) Summer: 24.2% (22) Autumn: 11.0% (10)
Time of sample collection	10:06 a.m. (51 min)
Frequency of hair washing per week	2.8 (1.6)
Regular use of hair products	Yes: 39.6% (36)
Hair color	Grey/white: 19.8% (17) Blonde/red: 34.9% (30) Brown/black: 45.3% (39) I do not know/missing: 5.5% (5)
Smoking	Yes: 12.1% (11)
Alcohol units per week^c^	2.1 (4)
Medications: hormone supplements	Yes: 9.9% (9)
BMI^d^	25.8 (4.6)
	Underweight (< 18.50): 2.2% (2) Normal (18.50 – 24.99): 41.8% (38) Overweight (25 – 29.99): 39.6% (36) Obese (≥ 30): 16.5% (15)
Shift work	Yes: 16.5% (15)
Physical activity score^e^	6.3 (6.6)
Sport	Less than 1 h a week: 35.2% (32) Regularly, 1–2 h a week: 44.0% (40) Regularly, 3–4 h a week: 15.4% (14) Regularly, more than 4 h a week: 5.5% (5)
Cups of coffee/tea per day	2.8 (1.9)

*BMI*  Body-Mass-Index; *HADS*  Hospital Anxiety and Depression Scale; *PDS*  Posttraumatic Diagnostic Scale; *PSQ-20 * Perceived Stress Questionnaire (20 item version); *SF-12*  Short Form-12 Health Survey; *SOMS*  Screening of Somatoform Disorders; *STAI*  State-Trait Anxiety Inventory (State Anxiety); *TQ*  Tinnitus Questionnaire.

^a^Education levels: low = elementary, secondary, or middle school; medium = high school or completed apprenticeship; high = university.

^b^Mean hearing threshold across all measured frequencies. Grading of hearing thresholds:^50^.

^c^Alcohol units consumed per week: one unit = 0.3 l beer or 0.2 l wine or shot glass of spirits.

^d^BMI classification:^52^.

^e^Physical activity score: number of days per week on which participants are physically active times the duration of the physical activity (1 = less than 10 min, 2 = 10–30 min, 3 = 30–60 min, 4 = more than 60 min).

#### Tinnitus pitch and loudness matching

Along with hearing thresholds, tinnitus matching data were collected from outpatient audiometric records. Prior to tinnitus matching, patients had been asked to indicate whether the tinnitus was currently audible, its location (left, right, bilateral), sound quality (more alike to pure tone or narrow-band noise), and approximate frequency range (high, medium, low). For the matching procedure, patients had been asked to indicate when a tone corresponded to their tinnitus, first in terms of frequency and then in terms of loudness. Depending on the specified frequency range (low, medium, high), three different frequencies had been initially played for comparison and were narrowed down to two different frequencies after a positive response; the final match had to be confirmed twice by the patients. Once the frequency had been identified, the loudness was adjusted in 1-dB steps starting at the hearing threshold; the final match had to be confirmed twice by the patients. Matched frequency (Hz) and loudness (dB) were averaged across both sides for bilateral tinnitus if measurements from both sides were available. The absolute matched tinnitus loudness was used as opposed to the relative sensation level (loudness above hearing threshold), as the hearing threshold was collected in 5-dB steps and tinnitus loudness in 1-dB steps, which limited the accuracy of the sensation level measure. However, the average hearing threshold was included in the analyses to control for the effect of hearing impairment on the absolute tinnitus loudness. Moreover, matched tinnitus loudness was chosen instead of subjective loudness ratings because the latter appeared to be more influenced by distress levels in our sample. For N = 25 patients, tinnitus matching was not possible either because the tinnitus was not audible at the time of measurement, its sound quality could not be captured by pure tones or narrow-band noise, or because its frequency exceeded the measurement range (> 10 kHz).

#### Additional tinnitus or hearing-related information

Additional information on tinnitus or hearing-related aspects were assessed by self-report: intermittent vs. constant tinnitus, tinnitus onset associated with stress, tinnitus influenced by stress, hyperacusis, and hearing aid use.

### Covariates

Covariates assessed by self-report included sociodemographic information (sex, age, marital status, education level, employment), hair care (frequency of hair washing per week, regular use of hair products, natural hair color), and health-related behavior (smoking, alcohol units consumed per week, hormone medications, cortisone medications, body-mass index (BMI), shift work, physical activity, sports, and cups of coffee/tea consumed per day). The use of cortisone medication was excluded as a predictor because only N = 4 gave an affirmative answer. Other recorded covariates were season and time of sample collection.

### Statistical analysis

Statistical analyses and plotting were performed using R (version 4.0.0)^51^. For data preparation, the package “tidyverse” was used; for correlations “Hmisc” and “corrplot”; for elastic net regression, “caret” and “glmnet”; and “RANN” for k-nearest neighbor imputation. Hair-cortisol concentrations were log-transformed to establish normal distribution.

#### Descriptive analyses and correlations

For sample description (Table 2), absolute numbers and frequencies are reported for categorical variables and mean values and standard deviations (SD) for numerical variables, psychometric questionnaires are categorized using the respective cut-offs, and hearing threshold and BMI values are categorized using defined WHO cut-offs^50,52^. For data exploration, Spearman correlations were calculated for continuous variables (missing values were deleted pairwise) and depicted in a correlation plot sorted by hierarchical clustering, see Supplementary Fig. S1.

#### Elastic net regression

Elastic net regression is a penalized linear regression method (a generalization of ridge and lasso regression) that performs shrinkage of correlated predictors and automatic variable selection^38,39^. Elastic net regression uses two tuning parameters: alpha (mixing parameter), ranging from 0 = ridge regression to 1 = lasso regression, and lambda (regularization parameter), which determines the overall strength of shrinkage/penalization (see glmnet^53^ vignette).

Two elastic net regression models with hair-cortisol and hair-BDNF levels as outcome variables were calculated. Predictors included psychometric questionnaire scores, matched tinnitus loudness and frequency, hearing threshold (audiometry), and other covariates; a total of 35 predictors for each model; see Table 2. Both outcome variables and predictors were standardized for better comparability of the results. For both outcomes, normality of residuals was met (visual check and Kolmogorov–Smirnov Test) and no predictors had zero or near-zero variance. Among predictors, one correlation of *r* > 0.75 (Spearman) was present: between tinnitus loudness and mean hearing threshold, *r* = 0.79, *p* < 0.001, N = 66. For both elastic net regression models, the dataset was randomly divided into a training dataset consisting of 70% of the data (N = 66 for cortisol; N = 63 for BDNF) on which the models were trained and a test dataset consisting of 30% of the data (N = 25 for cortisol; N = 24 for BDNF) on which the accuracy of the model predictions was tested. The data splitting ensured similar distributions of the outcome variables in the training and test datasets. The 70% to 30% split was chosen to obtain a sample size of N > 20 for the test data.

N-fold cross-validation was used to tune each elastic net model across 10 different lambda values and 10 different alpha values. The optimal model for each outcome was selected by minimizing the root mean square error (RMSE). Optimal regularization parameters for the prediction of cortisol were alpha = 0.4 and lambda = 0.28671; and for BDNF, alpha = 0.2, lambda = 0.14914; see Supplementary Fig. S2 and S3. Performance metrics (RMSE and R^2^) of the optimal models on the training and test datasets and estimated standardized coefficient effects are reported as results. In addition, variable importance (VI), a scaled ranking from zero to 100 based on the coefficient estimates, was used to group the effects by magnitude (in VI quartiles). Only effects with VI ≥ 50 were considered as main findings.

#### Imputation of missing values

Missing values on numeric predictors (27.5% on tinnitus matching data and 2.2% on SF-12) were imputed using k-nearest neighbor imputation. This method was chosen because it can handle different types of missing data. The remaining missing values on categorical predictors were 5.5% for natural hair color. Analyses were repeated in the subsample with complete tinnitus matching data (cortisol: N = 66, BDNF: N = 63) to assess possible influences of imputation.

## Results

**Figure 1:**
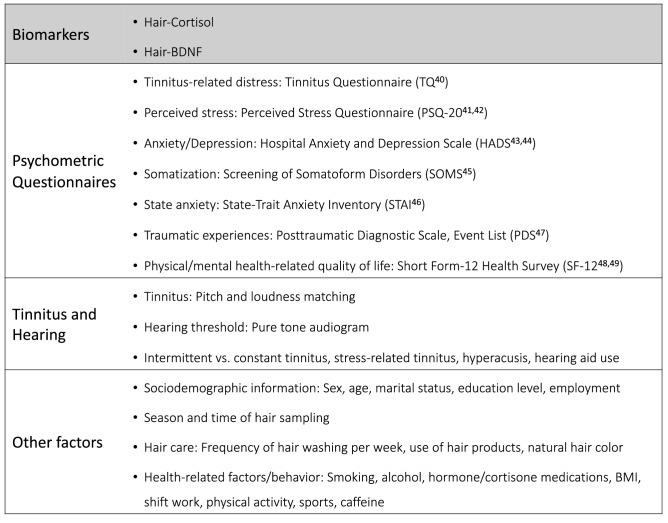
Overview of included study variables. Biomarkers (cortisol and BDNF measured in hair) were investigated as outcome variables while all other variables (psychometric questionnaires, tinnitus and hearing, covariates) were used as predictors.

**Figure 2:**
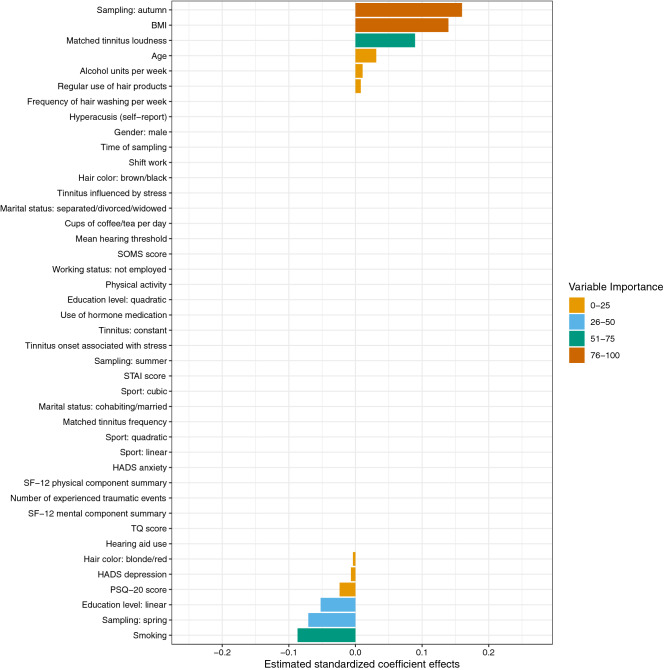
Estimated standardized coefficient effects by elastic net regression with n-fold cross-validation for the prediction of hair-cortisol in chronic tinnitus patients (training data: N = 66). *BMI*  Body-Mass-Index; *HADS*  Hospital Anxiety and Depression Scale; *PSQ-20*  Perceived Stress Questionnaire (20 item version); *SF-12*  Short Form-12 Health Survey; *SOMS* Screening of Somatoform Disorders; *STAI*  State-Trait Anxiety Inventory (State Anxiety); *TQ*  Tinnitus Questionnaire.

**Figure 3:**
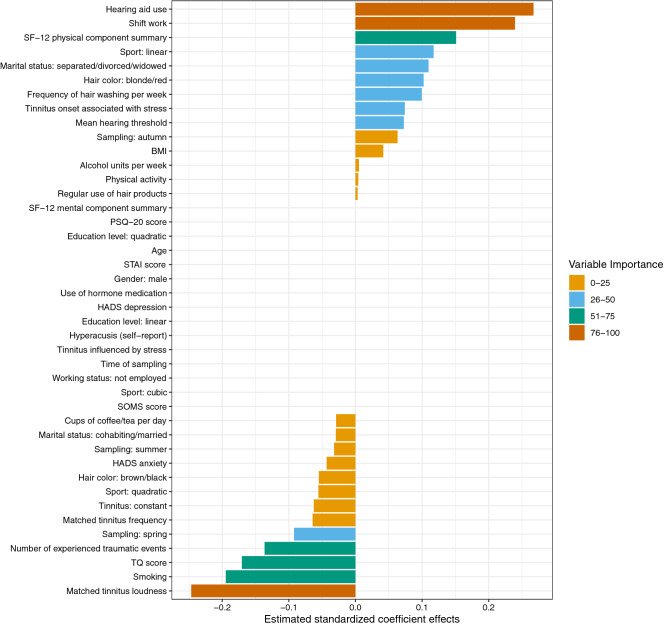
Estimated coefficient effects by elastic net regression with n-fold cross-validation for the prediction of hair-BDNF in chronic tinnitus patients (training data: N = 63). *BMI*  Body-Mass-Index; *HADS *Hospital Anxiety and Depression Scale; *PSQ-20*  Perceived Stress Questionnaire (20 item version); *SF-12*  Short Form-12 Health Survey; *SOMS *Screening of Somatoform Disorders, *STAI*  State-Trait Anxiety Inventory (State Anxiety); *TQ* Tinnitus Questionnaire.

The variables examined in this study are summarized in Fig. 1. All measurements (biomarker sampling, psychometric questionnaires, tinnitus pitch and loudness matching, pure tone audiometry, and collection of other information) were performed on the entire sample; missing values for tinnitus matching data and SF-12 were imputed (see “Methods”). Because the main aim of this study was to investigate associations of hair-cortisol and hair-BDNF with tinnitus-related and psychological factors while controlling for confounding influences, the two biomarkers were investigated as outcome variables using elastic net regression, whereas all other assessed variables were used as predictors. These analyses included N = 91 for hair-cortisol and N = 87 for hair-BDNF; results are reported below and shown in Figs. 2 and 3. In addition, the analyses were repeated in the subsample with complete tinnitus matching data (N = 66) to assess the influences of imputation.

### Sample description

The characteristics of the sample (N = 91) in terms of the assessed biomarkers, sociodemographic information, psychometric questionnaires, tinnitus/hearing as well as covariates are listed in Table 2. Approximately two-thirds of the sample were female (65.9%); on average, participants were middle-aged (M = 51.5, SD = 12); and most participants were cohabiting or married (53.8%), were employed (74.7%), and had medium (35.2%) to high (48.4%) levels of education; see Table 2.

### Prediction of hair-cortisol

For the prediction of hair-cortisol, the strongest predictive effects (VI > 75) were found for *sampling in autumn* and *BMI* (positive), followed by effects (VI > 50) for *matched tinnitus loudness* (positive) and *smoking* (negative). The elastic net regression model explained 6% of the variance in hair-cortisol in the training data (RMSE = 0.91, R^2^ = 0.06), and 10% of the variance in the test data (RMSE = 1.11, R^2^ = 0.10).

All estimated standardized coefficient effects and their grouping by VI are displayed in Fig. 2. In detail, positive associations with hair-cortisol levels were found for *sampling in autumn* (β = 0.160, VI = 100), *BMI* (β = 0.140, VI = 87.24), *matched tinnitus loudness* (β = 0.089, VI = 55.91), *age* (β = 0.031, VI = 19.52), *consumed alcohol units per week* (β = 0.011, VI = 6.66), and *regular use of hair products* (β = 0.008, VI = 5.00). Negative associations with hair-cortisol levels were found for *smoking* (β = −0.087, VI = 54.38), *sampling in spring* (β = −0.071, VI = 44.28), *education level (linear relationship)* (β = −0.052, VI = 32.71), *PSQ-20* (β = −0.024, VI = 14.86), *HADS depression* (β = −0.007, VI = 4.29), and *hair color: blonde/red* (β = −0.004, VI = 2.36). Note: The interpretation of coefficient effects obtained from elastic net regression is not different from ordinary least square multiple regression models. As an advantage, predictor effects can be directly compared in magnitude due to standardization.

### Prediction of hair-BDNF

For the prediction of hair-BDNF, the strongest effects (VI > 75) were found for *hearing aid use*, *shift work* (positive), and *matched tinnitus loudness* (negative); followed by (VI > 50) *smoking*, *TQ score*, *number of experienced traumatic events* (negative), and *SF-12 physical component summary* (positive). The elastic net regression model explained 25% of the variance in hair-BDNF in the training data (RMSE = 0.85, R^2^ = 0.25), and 28% of the variance in the test data (RMSE = 0.98, R^2^ = 0.28).

All estimated standardized coefficient effects and their grouping by VI are displayed in Fig. 3. In detail, positive associations with hair-BDNF levels were found for: *hearing aid use* (β = 0.267, VI = 100), *shift work* (β = 0.240, VI = 89.60), *SF-12 physical component summary* (β = 0.151, VI = 56.48), *sport (linear relationship)* (β = 0.117, VI = 43.89), *marital status: separated/divorced/widowed* (β = 0.110, VI = 41.05), *hair color: blonde/red* (β = 0.102, VI = 38.28), *frequency of hair washing per week* (β = 0.100, VI = 37.24), *tinnitus onset associated with stress* (β = 0.074, VI = 27.71), *mean hearing threshold* (β = 0.073, VI = 27.14), *sampling in autumn* (β = 0.063, VI = 23.64), *BMI* (β = 0.042, VI = 15.59), *alcohol units consumed per week* (β = 0.005, VI = 1.94), *physical activity* (β = 0.004, VI = 1.55), and *regular use of hair products* (β = 0.003, VI = 1.22). Negative associations with hair-BDNF levels were found for: *matched tinnitus loudness* (β = −0.247, VI = 92.22), *smoking* (β = −0.195, VI = 72.83), *TQ score* (β = −0.171, VI = 63.80), *number of experienced traumatic events (PDS)* (β = −0.136, VI = 51.02), *sampling in spring* (β = −0.092, VI = 34.49), *matched tinnitus frequency* (β = −0.064, VI = 24.07), *constant tinnitus* (β = −0.063, VI = 23.42), *sport (quadratic relationship)* (β = −0.056, VI = 20.81), *hair color: brown/black* (β = −0.055, VI = 20.56), *HADS anxiety* (β = −0.043, VI = 16.20), *sampling in summer* (β = −0.032, VI = 11.97), *marital status: cohabiting/married* (β = −0.029, VI = 11.02)*,* and *cups of coffee/tea per day* (β = −0.029, VI = 10.86).

### Models without imputation of tinnitus matching data

Additional analyses in the subsample with complete tinnitus matching data (N = 66), showed that without imputed matching data, tinnitus loudness was still identified as a predictor of hair-cortisol (β = 0.041, VI = 26.18) as well as of hair-BDNF (β = −0.005, VI = 3.11), but with lower variable importance.

## Discussion

This study was the first to analyze hair-cortisol and hair-BDNF levels in chronic tinnitus patients. We assessed their associations with tinnitus matching data, tinnitus-related distress, psychometric measures, and hearing threshold while controlling for potential confounders by using elastic net regression. Our results show that in chronic tinnitus patients, tinnitus loudness is associated with both increased hair-cortisol and decreased hair-BDNF levels, and tinnitus-related distress is associated with decreased hair-BDNF levels. This suggests that loud and distressing chronic tinnitus is linked to substantial long-term alterations of HPA axis function and adaptive neuroplasticity. Additional findings for hair-cortisol were positive effects of sampling in autumn and BMI, and a negative effect of smoking; for hair-BDNF, positive effects of hearing aid use, shift work, and higher physical health-related quality of life, and negative effects of smoking and previous traumatic experiences.

### Hair-cortisol: effect of tinnitus loudness

The finding that patients with higher perceived tinnitus loudness have higher hair-cortisol levels suggests that these individuals may show substantial HPA axis dysregulation, comparable to long-term effects reported in other chronic stress-exposed groups^22^. This complements previous studies analyzing salivary cortisol in tinnitus patients, which indicate a tendency for decreased HPA axis responsiveness^16–18^. In addition to increases in salivary cortisol and subjective stress levels after noise exposure, ^15^ found that patients with high tinnitus-related distress show an increase in tinnitus intensity, indirectly providing support for our finding. Regarding the conflicting findings on blood-cortisol levels in tinnitus, our result is consistent with the negative association of loudness reported by^20^, although in contrast, we did not observe any effect of tinnitus frequency. However, an important difference is that our result reflects long-term cortisol accumulation. Furthermore, no effect of the hearing threshold was identified, suggesting a hearing-independent effect of tinnitus loudness, apart from the limiting fact of generally normal hearing in our sample (63%).

Moreover, we did not find the expected relationship between tinnitus-related distress and hair-cortisol levels. This lack of association is consistent with findings on plasma cortisol in tinnitus by^19^. However, overall evidence regarding the relationship between hair-cortisol levels and subjective measures of perceived stress is inconsistent^22^. Mostly normal or mild perceived stress levels (77%) as well as predominantly normal depression (60%) and anxiety levels (47%) in our sample might explain the absence of psychological associations. Explained variance of hair-cortisol levels in the test data was comparatively low (10%), which may further indicate low modulatory influences on the HPA axis in our sample. Further indication for this assumption is the lack of an association between hair-cortisol and hair-BDNF levels, contrary to the observed negative association in the pilot hair-BDNF study in healthy stressed academics^37^.

### Hair-cortisol: effect of season, BMI, and smoking

Many confounding factors of hair-cortisol levels have been identified in the literature^54,55^. In our study, samples collected in autumn showed higher, and samples collected in spring showed lower hair-cortisol levels than samples collected in winter (reference category). This indicates seasonal variations in hair-cortisol concentrations, in line with previous studies^54–56^, yet the sample size was relatively small for both seasons (autumn: N = 10; spring: N = 15). In line with our result, a positive correlation between hair-cortisol and BMI is known from the literature^22,23^ and consistent with HPA axis dysregulation in obesity^57^. Two review articles concluded the absence of an association between smoking and hair-cortisol levels^22,23^, contrary to our negative result. The small smoking subsample size (N = 11) may have influenced this result. Overall, these findings highlight the importance to include confounding factors in hair-cortisol analysis.

### Hair-BDNF: effect of tinnitus-related distress and traumatic experiences

As expected, higher tinnitus-related distress was related to lower hair-BDNF, consistent with findings by^34^, where highly distressed tinnitus patients had lower serum BDNF levels than patients with mild distress. However, they found no difference between the high distress and the control group. Other previous studies measuring blood-BDNF in tinnitus did not find an association between tinnitus distress and BDNF levels in plasma^36^ or serum^33,35^. Our finding, however, should be more reflective of long-term effects than results from previous blood-BDNF measurements and is consistent with the expected negative effect of chronic stress on BDNF expression. This suggests hair-BDNF might be a useful biomarker to assess the clinical efficacy of treatments targeting tinnitus-related distress.

BDNF measured in hair may originate from blood circulation and from follicular epithelial cells^37^. However, the relative contribution of these mechanisms is unclear. For cortisol, the main mechanism is considered to be incorporation into growing hair cells by diffusion from the bloodstream^24,26^, and a recent study demonstrated, in agreement, that hair-cortisol represents circulating cortisol^58^. BDNF is widely expressed in the brain, especially in the hippocampus^30^, and 70–80% of circulating peripheral BDNF was found to originate from the brain^59^. Moreover, blood BDNF levels are positively correlated with hippocampal BDNF levels in animals^60^.

Further, animal studies showed that chronic stress leads to decreased BDNF (mRNA or protein) expression in the hippocampus^61–63^ as well as reduced volume of the hippocampus^64–66^. Depression is likewise associated with reduced BDNF in the periphery (serum:^67,68^) and reduced hippocampal volume^69^. Additionally, postmortem brain-tissue analysis showed reduced BDNF levels in the hippocampus of suicide subjects^70,71^.

Regarding tinnitus, both volume reductions^72,73^ and increases^74^ of the hippocampus have been reported, which were, however, unrelated to tinnitus duration and severity^73,74^. Conversely, ^75^ found a negative correlation between tinnitus distress and the left hippocampal surface. For a better understanding of neurobiological changes in chronic tinnitus, further studies on the associations of neuroplasticity changes, especially of the hippocampus, with tinnitus-related distress and hair-BDNF levels are needed. Findings on severity-dependent short-term memory and learning performance reductions in tinnitus^76^ are in line with the hypothesis of stress-related hippocampal neuroplasticity impairment in severe tinnitus.

BDNF has a complex role within the fear response circuitry^77^. Initially elevated serum BDNF levels in PTSD patients might be followed by long-term reduction^78^, consistent with our observed negative effect of traumatic experiences. Evidence further suggests that the BDNF Val66Met polymorphism, associated with deficient activity-dependent BDNF release^79^, might modulate the sensitivity to stress and trauma^77^. A recent study found that in tinnitus patients, the BDNF Val66Met polymorphism is associated with higher stress levels, higher levels of tinnitus-related distress, and activation/connectivity changes within a general distress network^80^. Whether our observed effects of tinnitus-related distress and traumatic experiences on hair-BDNF levels are influenced by the Val66Met polymorphism could be an interesting future research question.

### Hair-BDNF: effect of tinnitus loudness and hearing aid use

In contrast to the negative effect found for tinnitus loudness on hair-BDNF, previous blood-BDNF studies in tinnitus patients found no association between tinnitus loudness (tinnitus matching or visual analog scale) and serum or plasma BDNF levels^33,35,36^. We assume this discrepancy can be attributed to long-term effects only captured by BDNF measurement in hair. However, imputation of tinnitus matching data may potentially have led to an overestimation of the effect, which was considerably smaller in the subsample with complete matching data.

Evidence indicates that hearing aid use leads to neuroplasticity changes in the brain^81^. Thus, our finding might potentially suggest that hearing aid use counteracts detrimental chronic stress-related neuroplasticity effects in severe chronic tinnitus. However, as the number of hearing aid users was relatively small (N = 16) and we additionally observed a small positive effect of the mean hearing threshold, no conclusions can be drawn and further investigation in a larger-scale study is clearly needed.

### Hair-BDNF: effects of self-reported physical health-related quality of life, shift work, and smoking

Results of a meta-analysis indicate that regular exercise leads to subtle increases in peripheral BDNF levels^82^. In agreement, we found a positive effect of self-reported physical health-related quality of life and a small positive effect of sports activity on hair-BDNF levels, supporting the association between BDNF and physical health. However, despite a negative correlation between hair-BDNF and somatization, consistent with^37^, there was no effect of somatization when controlling for other influencing factors in the elastic net regression model. Other findings included effects of smoking and shift work but given the small number of smokers (N = 11) and shift workers (N = 15) and respective reliability issues, further research on these relationships is needed.

### Limitations

This study has some limitations. First, model performance for predicting hair-BDNF levels was higher (R^2^ = 0.28) than for hair-cortisol (R^2^ = 0.10). Since the models were tested on a different part of the data than on which they were built, performance estimates should be relatively robust. Therefore, it may be that hair-cortisol levels were generally more stable than hair-BDNF levels or influenced by other, unmeasured factors. However, generally normal/mild perceived stress and psychological symptom levels in our sample might have influenced this result. Second, some findings were based on small subsample sizes (seasonal effects, smoking, hearing aid use, shift work) and thus might have limited validity. Third, tinnitus matching data were collected from audiometric records, with an average time difference of 1.83 months (SD = 1.85) to the other measurements, which might have influenced the results. In addition, for N = 25 (27.5%) tinnitus matching could not be performed, and these missing values were imputed using k-nearest neighbor imputation. In models without imputation of tinnitus matching data, tinnitus loudness was still identified as a predictor for both hair-cortisol and hair-BDNF, but the effects were smaller, especially for hair-BDNF. Together with the lack of a correlation between hair-BDNF and tinnitus loudness, the validity of the effect of tinnitus loudness on hair-BDNF may be limited. Fourth, we did not include a control group in the present study as it did not reflect the research aim; therefore, no information is available on whether hair-cortisol and hair-BDNF levels were altered in our chronic tinnitus sample compared to healthy individuals. Based on our results, we expect higher hair-cortisol and lower hair-BDNF levels in patients with loud and distressing chronic tinnitus than healthy controls, but this assumption remains to be tested. Lastly, the investigated clinical sample was heterogeneous, and we observed a small predictive effect of constant vs. intermittent tinnitus on hair-BDNF levels. Accordingly, the observed effects may be particularly relevant for the subgroup of individuals with constant tinnitus, and future research might aim to investigate respective differences.

## Conclusions

In summary, we found that in chronic tinnitus patients, higher tinnitus loudness is associated with higher hair-cortisol and lower hair-BDNF levels, whereas higher levels of tinnitus-related distress are additionally associated with lower hair-BDNF levels. Effects were stronger for hair-BDNF than for hair-cortisol. Chronic tinnitus might be related to long-term changes in cortisol and BDNF expression, the strength of which may be moderated by perceived tinnitus loudness. High tinnitus-related distress and traumatic experiences appear to have additional detrimental effects on BDNF expression, whereas hearing aid use and high physical health-related quality of life appear beneficial. Results further highlight the importance of assessing confounders, like season, BMI, smoking, or shift work. The main implications of our findings are that cortisol levels measured in hair could serve as a biomarker of tinnitus loudness, whereas hair-BDNF levels might function as a presumably more sensitive biomarker of psychological or psychosomatic tinnitus-related distress in chronic tinnitus patients which could potentially be used to assess clinical treatment efficacy.

## Supplementary Information

Supplementary Figures.

## Data Availability

The datasets generated and analysed during the current study are available from the corresponding author on reasonable request.
