# Supplementary material for: Hair-cortisol and hair-BDNF as biomarkers of tinnitus loudness and distress in chronic tinnitus
Source: Sci Rep. 2022 Feb 4;12:1934. doi: 10.1038/s41598-022-04811-0 (PMC8817043; doi:10.1038/s41598-022-04811-0)
Supplement: Supplementary file 1 — Supplementary Figures. [file 41598_2022_4811_MOESM1_ESM.pdf]

Supplementary Material

Hair-Cortisol and Hair-BDNF as Biomarkers of Tinnitus Loudness and Distress in Chronic Tinnitus

Laura Basso[1], Benjamin Boecking[1], Patrick Neff[2,3,4], Petra Brueggemann[1], Eva M. J. Peters[5,6], Birgit Mazurek[1*]

[1]Tinnitus Center, Charité – Universitätsmedizin Berlin, Berlin, Germany
[2]Department of Psychiatry and Psychotherapy, University of Regensburg, Regensburg, Germany
[3]University Research Priority Program ‘Dynamics of Healthy Aging’, University of Zurich, Zurich, Switzerland
[4]Centre for Cognitive Neuroscience and Department of Psychology, University of Salzburg, Salzburg, Austria
[5]Psychoneuroimmunology Laboratory, Department of Psychosomatic Medicine and Psychotherapy, Justus-Liebig University Giessen, Giessen, Germany
[6]Psychosomatics and Psychotherapy, Charité Center 12 Internal Medicine and Dermatology, Charité – Universitätsmedizin Berlin, Berlin, Germany

**Corresponding author*

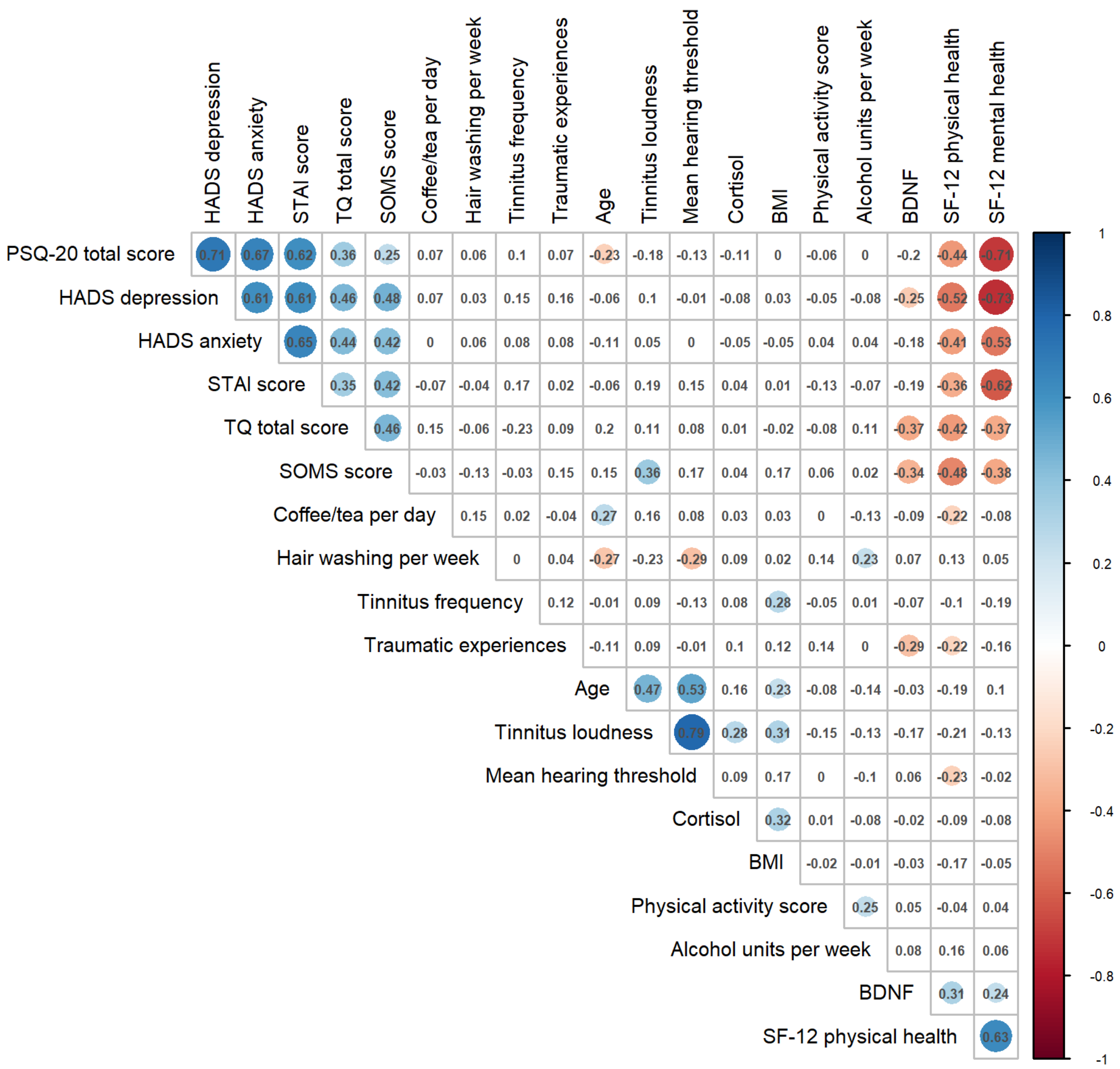

Supplementary Figure S1. Spearman correlation plot sorted by hierarchical clustering. Direction and strength of the correlations is indicated by color (blue=positive, red=negative). Only significant correlations (*p* < 0.05) are displayed; missing values were deleted pairwise (total N=91).

Abbreviations: BDNF = Brain-derived neurotrophic factor; BMI = Body-Mass-Index; HADS = Hospital Anxiety and Depression Scale; PSQ-20 = Perceived Stress Questionnaire (20 item version); SF-12 = Short Form-12 Health Survey; SOMS = Screening of Somatoform Disorders; STAI = State-Trait Anxiety Inventory (State-Anxiety); TQ = Tinnitus Questionnaire.

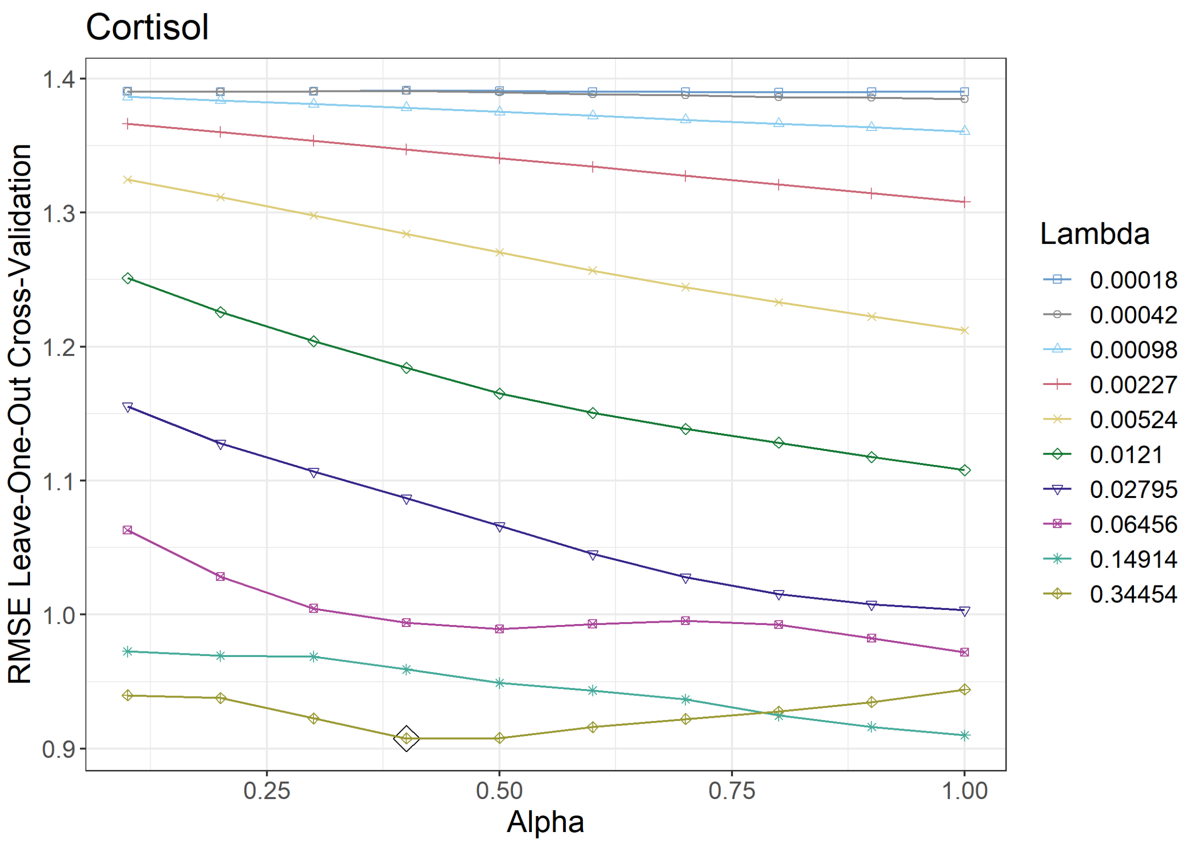

Supplementary Figure S2. Elastic net regularization (tuning across alpha and lambda values) with n-fold (leave-one-out) cross-validation for the prediction of hair-cortisol (training data: N=66). The diamond shape indicates the selected optimal model (smallest RMSE): alpha=0.4, lambda=0.28671.

Abbreviations: RMSE = root mean square error.

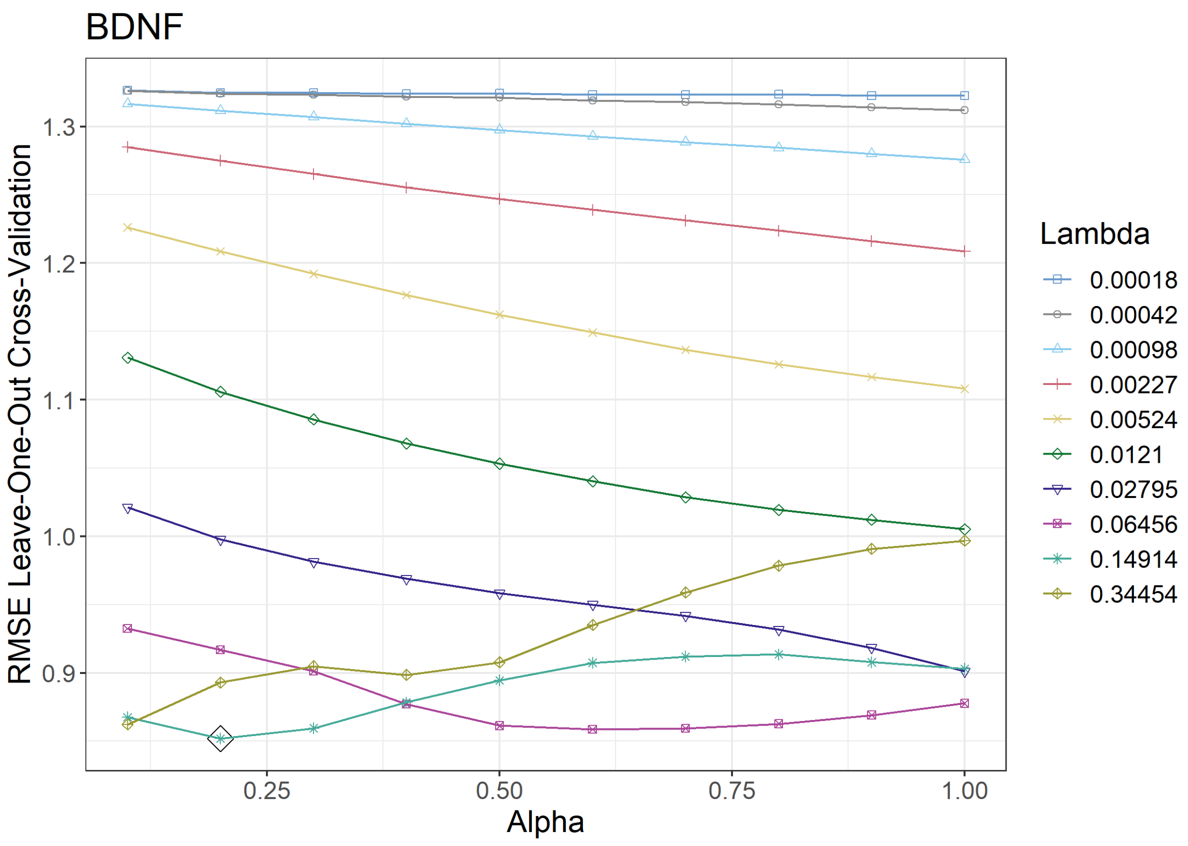

Supplementary Figure S3. Elastic net regularization (tuning across alpha and lambda values) with n-fold (leave-one-out) cross-validation for the prediction of hair-BDNF (training data: N=63). The diamond shape indicates the selected optimal model (smallest RMSE): alpha=0.2, lambda=0.14914.

Abbreviations: RMSE = root mean square error.
